# The chemical exfoliation phenomena in layered GaSe-polyaniline composite

**DOI:** 10.1186/1556-276X-8-29

**Published:** 2013-01-15

**Authors:** Olena Igorivna Aksimentyeva, Pavlo Yuriyovich Demchenko, Volodymyr Pavlovich Savchyn, Olexiy Alexandrovich Balitskii

**Affiliations:** 1 Department of Chemistry, Lviv Ivan Franko National University, Kyryla and Mefodiya Str, 6, Lviv, 79005, Ukraine; 2 Department of Electronics, Lviv Ivan Franko National University, Dragomanov Str. 50, Lviv, 79005, Ukraine

**Keywords:** Chemical exfoliation, Gallium selenide, Layered compound

## Abstract

To elucidate the nature of polyaniline (PANI)-GaSe mutual interaction, we carried out structural studies of nano-GaSe powders encapsulated by PANI, exploiting X-ray diffraction and high resolution transmission electron microscopy (HRTEM). Mechanically and ultrasonically dispersed GaSe crystals were mixed with aniline, which then underwent polymerization. After such treatment, GaSe nanocrystals (as shown by HRTEM) consist of few elementary monolayer sandwiches of hexagonal GaSe structure along the crystallographic axis *c* with a mean diameter of 9.2 nm. There was a significant expansion of interplanar distances (up to 0.833 nm) for all of the nanocrystals observed compared to 0.796 nm for the single crystals.

## Background

Two dimensional (2D) semiconductor nanocrystals fabricated in the plate-like form have been intensely investigated since the invention of single-layer graphene. The majority of binary compounds among them are either metal dichalcogenides (of molybdenum, vanadium, tungsten) or indium and gallium monochalcogenides. Gallium selenide with chemically passive selenium-terminated (11–20) surfaces has been applied as effective optical material for IR [1,2] and terahertz [3,4] ranges, termination layers in heterointerface fabrication [5-7], etc. Unique structure properties stand GaSe among materials suitable for production single layer 2D plates, even extracted and isolated from bulk. Although several groups have already succeeded in mechanical- [8,9], thermal-, and laser-induced [10] GaSe exfoliation, fabrication of free single sheet particles was found to be not an easy task. The properties of those GaSe foils are essentially substrate-dependent in the mechanical procedures, while higher temperature growth is accompanied by rolling of the sheets into more thermodynamically favorable [11] tubular 3D structures. Other successful attempts resulted in synthesis of colloidal single-layered nanoparticles in organic solutions [12-14] further underwent self-organization into more complicated structures [13,14] and fabricated by aqueous- or alcohol-based ultrasonification of GaSe powders [15,16]. The main problem in the application of such objects is synthesis and stabilization chemistry to be rather nonreproducible and hardly to be controlled as a rule. That usually results in unwanted electronic processes on surface-passivator boundaries and requires of the strongly binding ligands, preferably unreactive in respect to the ambient. Recent perspective [17] indicates that 2D plate-like nanoparticles (including those of GaSe) are excellent luminescent emitters due to the suppression of the absorption strengths into one electronic state in contrary to the band for a bulk material. Not long ago, we found that the mutual interaction of components in the hybrid composites containing GaSe and conducting polyaniline (PANI) polymer leads to an increased essential conductivity, UV shifting in GaSe luminescence spectra, plate-like particle formation, etc. [18]. The aim of the presented communication is an elucidation of the nature of the above-mentioned phenomena by means of structural studies of micro- (nano-) GaSe powders encapsulated by PANI, exploiting X-ray diffraction (XRD) and high resolution transmission electron microscopy.

## Methods

Aniline monomer, para-toluene-sulfonic acid, ammonium persulfate ((NH_4_)_2_S_2_O_8_) as oxidant were purchased from Aldrich Co., St. Louis, USA. Nanodispersed GaSe powder was obtained by mechanical milling of GaSe crystals, followed by ultrasonication in butanol. Both untreated GaSe single crystal plates and dried-in-vacuum GaSe nanopowders were used for the synthesis of hybrid nanocomposites with polyaniline.

Preparation of composites was carried out under conditions of oxidative polymerization of aniline under (NH_4_)_2_S_2_O_8_ in an aqueous medium in the presence of toluene sulfonic acid (TSA) as a doping and stabilizing agent. The method of obtaining the composite consists of several stages. Originally, the method was performed by dispersing of about 45 to 150 mg GaSe plates (such samples are further called PANI-GaSe sample) or GaSe powder with particle size of 60 to 80 nm (PANI-powdered GaSe sample) in a solution of surfactant 0.12 M TSA using ultrasonication for 30 min. Then, 0.205 g of monomer droplets was injected in the GaSe dispersion with continuous stirring, and after 10 min, the solution was added with 0.005 ml of 0.47 M solution of oxidant (NH_4_)_2_S_2_O_8_. The process was carried out at *T* = 293 K for 24 h. Finally, a dark dispersion of composite was isolated in the form of precipitate by centrifuging. For investigations, we took samples with inorganic component with 10 to 12% wt.

For transmission electron microscopy (TEM) and electron dispersive X-ray (EDX) characterization, a small amount of PANI-powdered GaSe sample (due to untransparency of bulk GaSe for electrons, PANI-GaSe sample was not suitable for TEM characterization) was diluted in anhydrous acetone and centrifuged; few drops of supernatant then were spread over a carbon-coated copper grip followed by drying (in a nitrogen atmosphere). That removes the traces of acetone and PANI capsules from GaSe nanocrystals. For X-ray diffraction measurements, GaSe-PANI and PANI-powdered GaSe samples were placed between two plastic slides.

XRD patterns were recorded in transmittance mode on a STOE STADI P spectrometer (STOE & Cie GmbH, Darmstadt, Germany) equipped with copper X-ray tube (the incident beam was passed through a germanium monochromator to produce K_*α*1_ radiation with a wavelength of 0.154056 nm). EDX and TEM images were obtained using a HR-TEM (Fei Technai G^2^ F20 S Twin microscope) operated at 300 keV.

## Results and discussion

**Figure 1:**
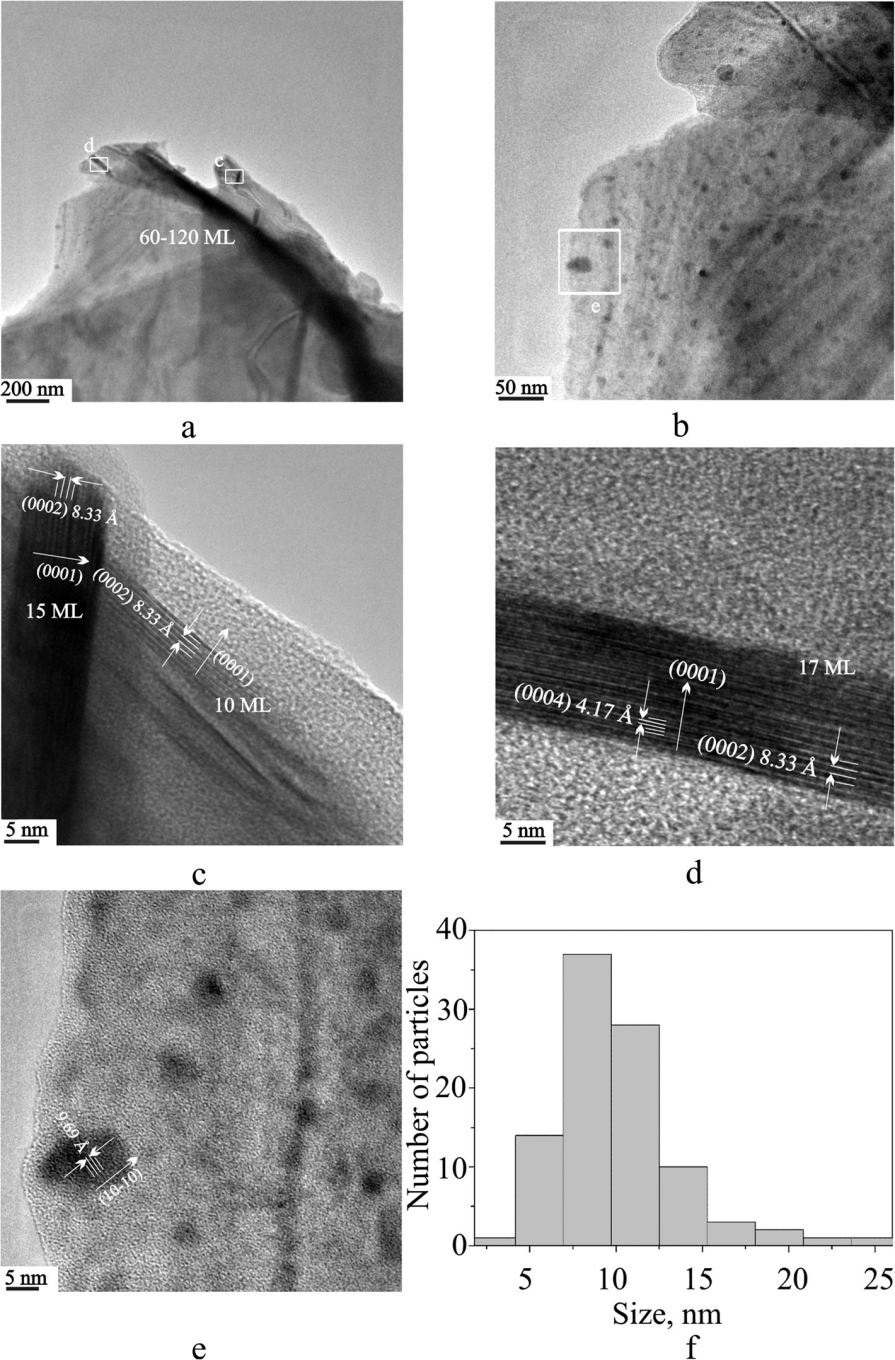
**TEM and HRTEM images of the nanoparticles. **Representative (**a**, **b**) TEM, HRTEM (**c**, **d**, **e**) of boxed areas (in **a**, **b**) images and size histogram made by counting over 100 particles from b TEM (**f**) of exfoliated by PANI–powdered GaSe nanoparticles. In the (**c**) and (**d**) images, the lattice planes could be attributed to the (0001) direction along the crystallographic *c* axis, while in the (**e**) image, to the (10–10) direction along the crystallographic *a* axis of hexagonal GaSe.

TEM images of PANI-powdered GaSe samples are presented in Figure 1. Expanded images (Figure 1a,b) demonstrate the presence (simultaneously with ultralarge microcrystals) of two types of nano-objects: thin stripes (up to few nanometers in height and up to 100 nm in length) and discs with rather broad diameter distribution (see Figure 1f). Beside the majority of particles are located inside 5 to 15 nm size region (mean size was estimated as 9.2 nm with 5.2 nm value of standard deviation), there are also several ultrasmall (less than 5 nm) and ultralarge (>20 nm) objects. The stripes and discs underwent atomic resolution on HR-TEM (Figure 1c,d,e), which magnifies boxed areas on Figure 1a,b. As shown by HRTEM (Figure 1c,d), the heights of such stripes consist of GaSe elementary sandwiches, packed along *с* crystallographic axis. It should be noted that there is an essential broadening of lattice plane spacing in this direction, yielding 0.833 nm (this value does not vary for many nanocrystals from different sample areas). Comparing with the same value for bulk GaSe material (0.796 nm for (0002) planes spacing), the *c* lattice parameter increased by about 4.4%. Some of the nanocrystals are so well resolved in order to obtain even fringes from higher (0004) planes with the same value of increasing *c* lattice parameter. The elementary tetralayers of GaSe structure along (11–20) direction are somehow bended by mechanical stresses, applied normally to above-mentioned direction on the whole particles (Figure 1c,d), but we did not observe any extended defects or elementary tetralayer fractures. The number of such monolayer (ML) per particles could vary from 10 to 20 (5 to 10 lattice parameters). The smaller particles (1 to 2 ML) during the interaction with electron beam, collinear to edges in plate-like particle geometry, simply do not effectively scatter electrons to make them visible by TEM, but were detected by optical measurements earlier [18] and as thin lattice resolved discs on Figure 1a,e. That is easily proved by comparing contrast of 10 and 15 to 17 ML particles in corresponding TEM images. The nature of disc-like particles is elucidated through analyzing Figure 1e. They are the same particles in Figure 1c,d, but their face plane oriented normally to the electron beam. We can also observe lattice fringed on one well oriented plane in respect to the electron beam. This time, the lattice spacing yields 0.969 nm, coinciding exactly with triple value of (10–10) lattice plane. From TEM and HRTEM images, we suggest that the particles are few-ML thick to observe lattice spacing similar to mechanically exfoliated GaSe flakes [9].

**Figure 2:**
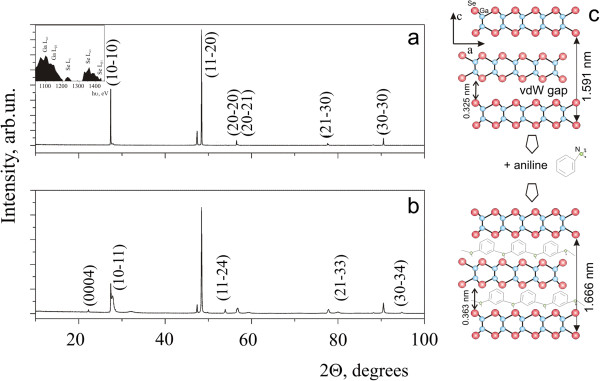
**XRD patterns, EDX spectrum and schematic presentation. **XRD patterns of PANI-GaSe sample (**а**), PANI-powdered GaSe sample (**b**), typical EDX spectrum of main elements (inset), and schematic representation of GaSe crystal structure (β-polytype, 2Hβ: space group *P*6_3_/mmc) interaction with PANI (**c**).

XRD patterns and EDX acquisition are presented in Figure 2. EDX (Figure 2, inset) confirms the initial stoichiometry of GaSe powders, predictably denying volatility losses (since we did not carry out any of the high temperature treatments). The other lines (not presented on expanded EDX spectrum) came both from organic components and TEM grid (copper, sulfur, nitrogen, oxygen, and carbon). After performing X-ray phase analysis, we can conclude that the formed object is a complex PANI-GaSe, a new chemical compound. While indexing PANI-GaSe XRD pattern (fitting up with the best texture model using WinCSD [19]), we came to the conclusion that the main phase in the sample is based on hexagonal GaSe (so-called β-polytype [20,21]), the spatial group P63/mmc with *a* = 3.75607 (10) and *c* = 16.15 (1) Å (already about 1.5% of *c* parameter increasing) with a dominant orientation (10–10) texture model. As shown in Figure 2a, there is also one additional diffraction peak in the interplanar distance (*d* = 1.917 Å) as well as some additional diffraction peaks with very low intensity (in particular, at *d* = 1.107 Å). Also, the applied texture model does not precisely describe the experimental diffractogram: the highest intensity reflection is (11–20), while according to the theoretical diffraction, it should be (10–10). The XRD of the PANI-powdered GaSe sample showed that during the milling, the crystal texture predictably decreases, and the diffractogram contains other diffraction reflections, characteristic for GaSe (Figure 2b). There is also the possibility of partial transition of β-GaSe polytype into the so-called ε-polytype GaSe (2Hα, space group P-6 m2), which shows in particular, the ratio of intensities of reflections (10–10) and (10–11). Note that the diffraction peak in the interplanar distance *d* = 1.917 Å persists. In fact, for that sample, any crystallographic refinement is generally unstable because of essential difference between the FWHM of reflections (they are either narrower or broader than theoretical). The simple calculations of angular positions of the reflections with third Miller index not equal to zero provide a *c* parameter very close to that one observed by TEM.

It is well known that gallium monoselenide crystal lattice (Figure 2c) consists of tetralayers: Se-Ga-Ga-Se-, bounded by the weak van der Waals forces. The interlayer distance between selenium-terminated sandwiches is approximately 3.25 Å. Due to this, it is possible to diffusively include polymeric chains of polyaniline between layers of Se-Se (the width of aniline molecule is about 2.8 Å in the thickest point of benzene ring). Obviously, polymerization results in much larger spatial hindrance of long PANI molecules when forming crystalline composite structures based on hexagonal GaSe. This changes the diffraction pattern which now does not accurately describe the prevailing model of orientation, creates the additional diffraction reflections, and is clearly elucidated by HRTEM. When utilizing the single-crystal plates, this composite phase is apparently saved, but there is simply hexagonal GaSe in contrary to the sample PANI-powdered GaSe. As it was mentioned earlier [18,22], powdered (i.e., fractured) GaSe samples exhibit numerous extended defects-cleavage stairs on the surface. The aniline molecules diffuse through them more effectively, filling van der Waals gap of particles (Figure 2c). That forms few ML composite particles based on GaSe-PANI compounds. As we have not observed any lattice fringes that exceeded 8.33 Å for (0002) GaSe crystal planes, we conclude that this is a critical parameter of GaSe-PANI composites based on GaSe crystal structure. Further hindrance of PANI in the van der Waals gap unambiguously leads to the formation of free isolated particles. The low-temperature synthesis procedure and the presence of PANI on GaSe edges permit to avoid thermodynamically preferable rolling of plane-like particles into tubular, onion [10], or belt-like [23] 3D structures.

## Conclusions

Few ML gallium selenide-PANI nanoparticles have been synthesized using chemical exfoliation method. They possess highly crystalline structure similar to bulk GaSe, but with essential broadening of interplanar distances. The obtained few-nanometer thick disk-like flakes possess broad diameter distribution with average value of 9.2 nm. These results enlighten new frontiers for the development of optical nanomaterials. They extend the fabrication techniques such as mechanical and thermal procedures, not suitable either for formation of size controlled or plate-like particles and organic syntheses, drastically affected by stabilizing ligands.

## Competing interest

The authors declare that they have no competing interests.

## Authors’ contributions

OIA carried out the synthesis of nanocomposites. PYuD participated in XRD measurements and structure refinements. VPS supervised the work and finalized the manuscript. OAB designed the experiment, participated in the structural investigation and drafted the manuscript. All authors read and approved the final manuscript.

## Authors’ information

OIA is currently the leading researcher of Physical Chemistry Department. PYuD is working as a senior researcher of Inorganic Chemistry Department. VPS and OAB are professor and associate professor, respectively, of Semiconductor Physics Department. All authors are from the Lviv Ivan Franko National University.
